# Follow-up evaluation of long COVID syndrome in patients with SARS-CoV-2 infection

**DOI:** 10.1590/0037-8682-0046-2025

**Published:** 2025-08-08

**Authors:** Sezin Hoşgel Sevdimbaş, Ayşe Seza İnal, Ferit Kuşcu, Behice Kurtaran, Aslıhan Candevir, Yeşim Taşova, Süheyla Kömür

**Affiliations:** 1 University of Health Sciences Adana City, Training and Research Hospital, Department of Infectious Diseases and Clinical Microbiology, Adana, Turkey.; 2 Cukurova University Faculty of Medicine, Department of Infectious Diseases and Clinical Microbiology, Adana, Turkey.

**Keywords:** long COVID, Post-COVID-19 Functional Status, SF-36

## Abstract

**Background:**

Long COVID, which refers to persistent symptoms following acute COVID-19, is being increasingly reported. However, available data regarding its prevalence and characteristics are limited. This study was conducted to evaluate the occurrence and presentation of long COVID in patients with COVID-19 who were followed up in outpatient, ward, or intensive care settings.

**Methods:**

This study included patients who were diagnosed with COVID-19 at least four weeks prior to the start of the study. The patients underwent symptom assessment at baseline and 1, 3, 6, and 12 months post-infection. Functional status was evaluated using the Post-COVID-19 Functional Status (PCFS) Scale, and quality of life was assessed using the SF-36 Health Survey questionnaire.

**Results:**

A total of 134 patients (71 males [54.2%]), with a mean age of 43.7 (15.3) years, participated in this study. The distribution of patients across care settings was as follows: 51.1% outpatients, 35.9% in wards, and 13% in intensive care units (ICUs). Fatigue during exertion was the most frequently reported long COVID symptom. Patients treated in ICUs experienced a higher burden of long COVID symptoms than those treated in outpatient or ward settings. Furthermore, patients treated in ICUs and wards had a significantly poorer quality of life and functional status than the outpatients.

**Conclusions:**

Long COVID poses a significant ongoing health concern, particularly for patients who require intensive care during acute COVID-19. Vigilant monitoring of long-term sequelae, particularly those that affect quality of life and functional status, is crucial for individuals recovering from COVID-19.

## INTRODUCTION

Severe acute respiratory syndrome coronavirus 2 (SARS-CoV-2), which causes the novel coronavirus disease (COVID-19), was first detected in China in December 2019. The virus rapidly spread worldwide, causing a global pandemic, with several new variants identified since the first case was reported^1^. Although many patients with COVID-19 recover within 2-3 weeks, long-term symptoms have been observed in some patients^2^. Accumulating evidence from several studies is steadily increasing our knowledge of the long-term effects of COVID-19. Data obtained during the SARS epidemic in 2003 and the Middle East Respiratory Syndrome epidemic in 2012, which were caused by other coronaviruses, indicate that some patients with either disease exhibited long-term symptoms such as impaired pulmonary and physical functions, decreased quality of life, and emotional stress after the acute disease period. It has been proposed that patients with COVID-19 may experience long-term symptoms^3^. However, the number of patients affected by late sequelae of acute COVID-19 remains unknown. Persistent symptoms of COVID-19 are more common in women, and the risk of having these symptoms is linearly correlated with age. Notably, these long-term symptoms seem to occur regardless of the initial severity of the infection and often affect more than one organ system^4^. 

There is no standardized terminology for the period after acute COVID-19 in which clinical findings do not fully improve. However, several names, such as long COVID, post-COVID-19, and post-acute COVID-19, have been used in previous reports^5^. Given that the absence of globally standardized terminologies and clinical definitions poses difficulties in epidemiological reporting, research, clinical management of affected patients, and treatment development, an international panel was established under the leadership of the World Health Organization (WHO) to clearly define this post-COVID period and its associated symptoms. The panel defined post-COVID-19 symptoms as those that occur in individuals with a history of probable or confirmed SARS-CoV-2 infection, usually occurring three months after the onset of COVID-19, with symptoms that cannot be explained by an alternative diagnosis lasting for at least two months. Common symptoms include fatigue, shortness of breath, and cognitive dysfunction, which often impact daily functioning^4^. 

Although knowledge of the long-term effects of COVID-19 has significantly increased worldwide, data from low- and middle-income countries are limited^6^. In Turkey, where 17,004,713 COVID-19 cases have been confirmed, a high frequency of long-term complications is expected^7^. This aim of this study was to advance the understanding of COVID-19 by evaluating the prevalence and characteristics of long COVID-19 syndrome in patients who were diagnosed with COVID-19 and followed up in outpatient clinics, hospital wards, or intensive care units (ICUs). In addition, we aimed to assess the patients’ functional limitations using the Post-COVID-19 Functional Status (PCFS) scale, which has been validated for the Turkish population, and their quality of life using the Short Form 36 (SF-36) Health Survey questionnaire.

## METHODS

### Study population

A total of 131 patients who were diagnosed with COVID-19 through PCR testing of nasopharyngeal swab samples and followed up at the Department of Infectious Diseases and Clinical Microbiology of the Cukurova University Faculty of Medicine between 2020 and 2021 were included in this study. We did not routinely perform viral sequencing to identify SARS-CoV-2 variants. However, all the patients included in this study were infected between late 2020 and 2021, and the predominant variants in Turkey during this period were the Alpha (B.1.1.7) and Delta (B.1.617.2) variants^8^ ^,^ ^9^. 

Patients who expressed their willingness to participate in this study at four weeks post-diagnosis were included for analysis. Patients younger than 18 years; those unable to complete the study forms due to dementia, learning disabilities, or other cognitive or communication disorders; and patients who had COVID-19 for less than four weeks were excluded from the study. Informed consent was obtained from all patients who agreed to participate in the study. This study was approved by the Çukurova University Faculty of Medicine Clinical Research Ethics Committee (decision no: 24, date: February 12, 2021). 

A structured questionnaire was designed to collect information on the patients’ current health status and persistent symptoms during the post-COVID period. All eligible patients were contacted for participation in this study. All the patients who were included in the study were personally followed up by the first author for one year. Patients who were able to visit the hospital were invited to participate in in-person interviews. Those who could not visit the hospital were required to complete questionnaires administered via telephone calls. The interviews were conducted by the first author to ensure consistency and reliability of data collection. The questionnaire collected demographic data, including age, sex, and the presence of comorbidities such as diabetes mellitus, hypertension, cardiovascular disease, chronic obstructive pulmonary disease, asthma, malignancy, chronic renal failure, obesity, and HIV infection. The presence and nature of long COVID symptoms were also assessed. Patients were grouped and evaluated according to the level of care they required after COVID-19 diagnosis: outpatient, ward, or ICU monitoring. length of hospital stay was recorded for patients who required care in the ward or ICU. Each patient underwent a comprehensive assessment of the COVID-19 symptoms, including fever, weakness, fatigue during exertion, cough, shortness of breath, myalgia, arthralgia, palpitations, headache, sleep disturbance, forgetfulness, difficulty concentrating, loss of appetite, loss of smell, loss of taste, diarrhea, skin findings, and visual impairment. The patients were also asked about any other complaints they had experienced. These symptoms were assessed and recorded at the time of diagnosis and at the 1-, 3-, 6-, and 12-month follow-up timepoints. At the time of the conceptualization and design of our study methodology (early 2021), no internationally standardized definition of Post-Acute Sequelae of COVID-19 (PASC) had been established. Therefore, we based our inclusion criteria on the prevailing clinical approach used at the time, in which persistent symptoms experienced beyond four weeks after acute infection were considered indicative of post-COVID syndrome^10^. The PCFS scale was used for assessment of functional status at 1, 3, 6, and 12 months post-diagnosis. The SF-36 survey was administered to assess quality of life at the same follow-up timepoints. Patient outcomes were documented as recovery or death (exitus). 

### Post-COVID-19 Functional Status Scale

The PCFS scale, which was developed by Klok et al., is an ordinal scale designed to assess functional recovery at four and eight weeks after hospital discharge and functional sequelae at six months. It comprises six grades ranging from 0 (no functional limitations) to 5 (death). Grade D is assigned to patients who died. Grade 1 and above indicate increasing degrees of symptoms, pain, or anxiety. The PCFS scale classifies functional status limitations as follows: grade 0, no functional limitation; grade 1, negligible functional limitation; grade 2, mild functional limitation; grade 3, moderate functional limitation; grade 4, severe functional limitation; and grade 5, Death^11^.

### Short Form 36 Health Survey

The SF-36 Health Survey is a generic quality-of-life instrument that used for broad assessment of health status. It consists of 36 items and evaluates health status based on the following eight subscales: physical function (10 items), social function (2 items), role limitations due to physical function (4 items), role limitations due to emotional problems (3 items), mental health (5 items), energy/vitality (4 items), pain (2 items), and general perception of health (5 items). Instead of providing a single total score, the scale provides a total score for each subscale separately. The total points range from 0 to 100, with 0 indicating poor health and 100 indicating good health^12^.

### Statistical analysis

Statistical analyses were performed using Statistical Package for the Social Sciences (SPSS) version 25.0. Categorical variables are summarized as frequencies and percentages or as means and standard deviations (medians and minimum-maximum values) as appropriate. Categorical variables were compared using the chi-square and Fisher’s exact tests. The Shapiro-Wilk test was used to assess normality in the distribution of continuous variables, whereas the Kruskal-Wallis test was used to compare non-normally distributed continuous variables. A post hoc analysis was conducted using Tamhane’s T2 test to identify differences between specific groups. Statistical significance was set at p < 0.05. 

## RESULTS

A total of 131 patients were included in this study. Of the 131 patients, 71 (54.2%) were male. The mean age of the patients was 43.7 (15.3) years (median: 45; range: 18-83). Eighty-one (61.8%) patients had comorbidities, and the most prevalent was hypertension (23, 17.6%), followed by cardiovascular disease (18, 13.7%) and diabetes mellitus (15, 11.5%). The mean age of patients treated in the ICU was significantly higher than that of patients treated in the general ward or as outpatients (p < 0.001). The ICU and general ward groups had a higher proportion of male patients and a higher prevalence of comorbidities such as diabetes (p = 0.029), hypertension (p =0.028), and cardiovascular disease (p <0.001) than the outpatient group (Table 1). Evaluation of patient symptoms revealed that 99.2% of the patients experienced at least one symptom at the time of diagnosis. This rate progressively decreased to 87% at 1 month, 68.7% at 3 months, 55.6% at 6 months, and 48.9% at 12 months. In addition, evaluation of the patients’ symptoms revealed distinct temporal patterns. At the time of diagnosis, the most prevalent symptoms were weakness and fatigue (86.3%), easy fatigue during exertion (70.2%), cough (69.5%), and myalgia (71.0%). Easy fatigue during exertion (74.8%) and weakness (56.5%) remained the most common complaints in the first month post-COVID-19 diagnosis. Notably, easy fatigue during exertion persisted as the primary symptom throughout the follow-up period, affecting 50.4% of patients at 3 months, 38.2% at 6 months, and 36.6% at 12 months. At the time of diagnosis, the outpatients had a significantly lower frequency of fever (p = 0.021) and dyspnea (p = 0.002) than those treated in the ward or ICU. Conversely, outpatients exhibited a higher frequency of loss of smell (p <0.001) and loss of taste (p =0.010) than the other patient groups. Analysis of COVID-19 symptoms at 1, 3, 6, and 12 months post-diagnosis revealed that patients initially treated in ICUs experienced significantly higher rates of persistent symptoms than those treated in general wards or as outpatients. 

**TABLE 1:** Baseline characteristics of the patient groups.

	Total	Outpatient (n=67)	General ward (n=47)	Intensive care unit (n=17)	p value^a^
	n (%)	n (%)	n (%)	n (%)	
Sex					
Female	60 (45.8)	44 (65.7)	14 (29.8)	2 (11.8)	<0.001**
Male	71 (54.2)	23 (34.3)	33 (70.2)	15 (88.2)	
Comorbidities	81 (61.8)	30 (44.8)	36 (76.6)	15 (88.2)	<0.001**
Diabetes mellitus	15 (11.5)	3 (4.5)	8 (17.0)	4 (23.5)	0.029*
Hypertension	23 (17.6)	6 (9.0)	13 (27.7)	4 (23.5)	0.028*
Cardiovascular disease	18 (13.7)	3 (4.5)	8 (17.0)	7 (41.2)	<0.001**
COPD/Asthma	14 (10.7)	3 (4.5)	5 (10.6)	6 (35.3)	0.001**
Obesity	8 (6.1)	3 (4.5)	3 (6.4)	2 (11.8)	0.531
Chronic renal failure	2 (1.5)	1 (1.5)	1 (2.1)	-	0.828
HIV infection	15 (11.5)	13 (19.4)	1 (2.1)	1 (5.9)	0.013*
		**Outpatient (n=67)**	**General ward (n=47)**	**Intensive care unit (n=17)**	**p-value** ^b^
		**Mean (SD) Med (Min-Max)**	**Mean (SD) Med (Min-Max)**	**Mean (SD) Med (Min-Max)**	
Age	43.7 (15.3)	36.4 (13.2)	48.9 (13.2)	58.0 (12.1)	<0.001**
	45 (18-83)	31 (18-76)	51 (20-83)	57 (32-81)	

*p-value <0.05, **p-value<0.001. Mean, average; **SD:** standard deviation; **Med:** median, **Min:** minimum; **Max:** Maximum; **a:** chi-square and fisher exact test, **b:** Kruskal Wallis test.

**FIGURE 1:**
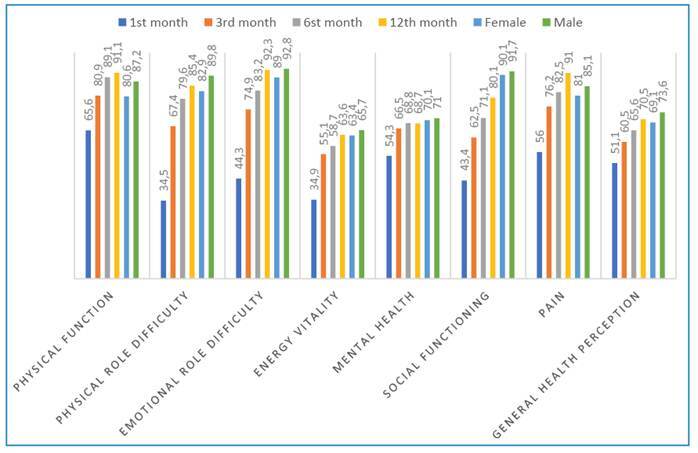
Comparison of the SF-36 subscale scores of the patients at 1, 3, 6, and 12 months post-COVID-19 diagnosis with the normal values for the general Turkish population.

The detailed results are presented in Supplementary Table 1. The distribution of patient PCFS scale scores across multiple timepoints, including the pre-COVID-19 period, time of diagnosis, and at 1, 3, 6, and 12 months post-diagnosis, are summarized in Supplementary Table 2. Although 71.8% of the patients reported no or negligible functional limitation (PCFS scores 0 or 1) in the pre-COVID period, this percentage declined sharply to 14.6% one month after diagnosis. Gradual improvement was observed at the subsequent follow-up timepoints, with 44.2% reporting no or minimal limitation at six months and 50% at 12 months. However, complete recovery to the pre-COVID-19 functional status was not achieved in all patients. The outpatient group showed significantly higher frequencies of no functional limitation (grade 0) in the pre-COVID period, at the time of diagnosis, and at 12 months post-COVID (all p < 0.001) than those treated in the wards or ICUs. In contrast, patients treated in wards or ICUs exhibited higher rates of severe limitation (grade 4) at one month (p < 0.001) and moderate-to-severe limitation (grades 3 and 4) at three months post-diagnosis (p = 0.002) Supplementary Table 2. Comparison of the eight SF-36 subscale scores at 1 and 3 months post-diagnosis to the normative data of the general Turkish population revealed that the patients had significantly lower scores across all domains. However, significant improvements in these scores were observed at 6 and 12 months post-diagnosis (Figure 1). Analysis of the SF-36 scale scores at one month post-diagnosis revealed that patients initially treated in the ICU had significantly lower scores in the physical function (p <0.001), physical role difficulty (p =0.001), energy vitality (p =0.028), social functioning (p =0.001), and general health perception (p <0.001) subscales than those treated in general wards or as outpatients. The differences in physical function (p =0.001), physical role difficulty (p =0.025), and social functioning (p =0.031) persisted at 12 months, with the ICU patients reporting lower scores. Delta values of the differences between the scale scores recorded in the first and twelfth month were calculated to assess changes in quality of life over time (Table 2). The results demonstrated that patients in the ICU group experienced greater improvements in physical function (p-value =0.001), difficulties in emotional roles (p-value=0.049), and general health perception (p-value <0.001) than the other groups.

**TABLE 2:** Differences (delta values) in the scale scores of the patient groups between the first and twelfth months post-diagnosis.

	1^st^ month	12^th^ month	Delta
	Mean (SD)	Mean (SD)	(12^th^ -1^st^ )
Physical function			
Outpatient (n=67)	77.2(24.1)	93.9(11.6)	17.1(21.5)
General ward (n=47)	63.7(30.7)	89.5(14.0)	24.9(26.5)
Intensive care unit (n=17)	25.6(37.3)	84.1(20.6)	58.5(35.6)
p-value	<0.001**	0.001**	0.001**
**Difficulties in physical roles**			
Outpatient (n=67)	44.8(44.1)	90.8(24.4)	46.2(42.7)
General ward (n=47)	30.3(41.7)	84.3(32.3)	52.9(48.9)
Intensive care unit (n=17)	5.88(24.3)	67.6(43.1)	61.8(45.2)
p-value	0.001**	0.025*	0.758
**Difficulties in emotional roles**			
Outpatient (n=67)	44.8(44.0)	92.8(23.9)	46.7(48.2)
General ward (n=47)	51.1(49.1)	90.7(29.4)	36.4(52.4)
Intensive care unit (n=17)	23.5(43.7)	94.1(24.3)	70.6(46.9)
p-value	0.106	0.907	0.049*
**Energy/vitality**			
Outpatient (n=67)	38.6(21.9)	63.8(16.3)	24.9(25.1)
General ward (n=47)	33.8(24.9)	63.1(15.7)	27.8(23.9)
Intensive care unit (n=17)	23.2(25.2)	64.1(9.7)	40.9(23.7)
p-value	0.028*	0.607	0.064
**Mental health**			
Outpatient (n=67)	54.9(19.5)	68.2(13.8)	12.2(8.6)
General ward (n=47)	57.4(23.9)	69.8(11.5)	10.7(24.1)
Intensive care unit (n=17)	43.8(26.6)	68.2(11.1)	24.5(26.5)
p-value	0.121	0.877	0.060
**Social functioning**			
Outpatient (n=67)	50.4(25.6)	83.3(18.1)	31.5(32.5)
General ward (n=47)	41.6(37.1)	78.2(19.1)	35.7(36.5)
Intensive care unit (n=17)	20.6(28.6)	72.8(13.4)	52.2(29.4)
p-value	0.001**	0.031*	0.061
**Pain**			
Outpatient (n=67)	55.5(32.7)	91.9(14.8)	36.3(31.8)
General ward (n=47)	55.9(35.6)	88.1(22.5)	31.3(36.4)
Intensive care unit (n=17)	58.5(34.8)	95.3(13.4)	36.8(32.1)
p-value	0.968	0.245	0.335
**General health perception**			
Outpatient (n=67)	57.5(21.1)	72.9(13.9)	14.3(18.1)
General ward (n=47)	48.4(17.5)	68.9(17.5)	19.9(18.1)
Intensive care unit (n=17)	33.5(19.2)	65.3(14.7)	31.8(17.0)
p-value	<0.001**	0.078	<0.001**

*p-value<0.05, **p-value <0.001, Kruskal-Wallis test.

## DISCUSSION

The results of this study demonstrated that the patients with COVID-19 who were treated in hospital wards or ICUs had a higher incidence of long COVID and experienced greater impairment in quality of life and functional status than those treated as outpatients. These findings contribute to the growing body of evidence on the impact of persistent post-COVID-19 symptoms on patients’ well-being and daily functioning. Notably, this is the first study on the assessment of quality of life using the SF-36 Health Survey and functional status using the PCFS scale in Turkish individuals with long COVID. COVID-19-related symptoms are heterogeneous; similarly, a wide variety of post-COVID-19 symptoms have been reported. In the present study, 87% of the patients had at least one symptom in the first month, 68.7% in the third month, 55.6% in the sixth month, and 48.9% in the twelfth month. In an Italian study that included 143 patients evaluated for persistent symptoms 60 days after discharge, only 18 (12.6%) reported no COVID-19-related symptoms; 32% had one or two symptoms, and 55% had three or more symptoms. The most common symptoms reported by the patients were fatigue (53.1%), shortness of breath (43.4%), arthralgia (27.3%) and chest pain (21.7%)^2^. A meta-analysis of 21 studies that included a total of 47,910 patients with long COVID highlighted 55 long-term symptoms associated with COVID-19. In the study, fatigue (58%), headache (44%), attention deficit (27%), hair loss (25%), and shortness of breath (24%) were the most common symptoms that persisted after recovery from COVID-19^13^. In the present study, the most common symptoms experienced by the patients in the first month post-COVID-19 were fatigue during exertion (74.8%), fatigue (56.5%), myalgia (34.4%), arthralgia (27.5%), and dyspnea (26.7%). These five symptoms were the most prevalent during the initial phase of recovery. Notably, fatigue, weakness, and fatigue during exertion persisted as the most commonly reported symptoms at the 3-, 6-, and 12-month follow-up timepoints. Huang et al. and Peghin et al. reported the results of patient evaluations at an average of six months after acute COVID-19. Huang et al. evaluated 1733 patients with COVID-19 at an average of six months from symptom onset and found that 76% of the patients still experienced ongoing symptoms, with the symptom rate being even higher among those with severe disease^14^. Peghin et al. reported a prevalence of 40.2% for persistent COVID-19 symptoms in their 6-month follow-up study, which included 599 patients with COVID-19 ranging from asymptomatic to severe and critical. The authors found that dyspnea, fatigue, and neurological symptoms were significantly associated with the severe disease, whereas the loss of taste, smell, and other sensory disorders were significantly associated with mild illness^15^. Consistent with previous reports, patients in the present study were assessed at 1, 3, 6, and 12-months post-diagnosis. 

Evaluation of symptoms at each follow-up timepoint showed that symptoms such as fatigue during exertion, general weakness/fatigue, dyspnea, sleep disturbance, forgetfulness, and palpitations were significantly more prevalent in the ICU patient group than in the outpatient and ward groups. Peghin et al. reported that taste and smell disorders at diagnosis and at three months post-diagnosis were significantly more common in the outpatient group than in other groups (p <0.05)^15^ One possible explanation for these differences is the higher prevalence of comorbidities such as diabetes, hypertension, and cardiovascular disease in patients treated ICUs and wards. Previous studies have shown that chronic conditions are associated with prolonged recovery and an increased risk of post-COVID complications^16^. COVID-19 has affected the short- and long-term quality of life of millions of people worldwide. Quality of life, an important concept in evaluating the impact of disease on an individual, can be affected by a combination of psychological, social, emotional, physical, and mental health factors^17^. The SF-36 test is widely used for assessment of quality of life. The results of the present study indicated that some of the SF-36 subscales were negatively affected by COVID-19, with the seriously ill patients in the ICU group having lower scores than those in the outpatient and ward groups. This finding aligns with those of an observational cohort study conducted in the United States, in which 62 patients who recovered from COVID-19 were evaluated using the SF-36 scale. The participants were categorized into mild, moderate, or severe disease groups based on their oxygen requirements during the acute phase of the disease. The patients were assessed using the SF-36 scale at least 15 days after SARS-CoV-2 PCR positivity. Analysis of their SF-36 subscale scores showed that the patients with moderate and severe disease had significantly lower scores in the physical function, role limitation due to physical health, energy/vitality, social functionality, and general health subscales than patients with mild disease. The results of the study emphasized that severe and moderate COVID-19 have a greater impact on quality of life than mild disease, particularly on physical function, role limitations due to physical health, energy/fatigue, pain, social function, and general health^18^. In another prospective observational study that included 124 patients, health status was assessed at the 3-month timepoint after COVID-19 diagnosis. Patients were classified into mild, moderate, or critical disease groups, and their health statuses were evaluated using both the SF-36 scale and the Nijmegen Clinical Screening Instrument. The results revealed that the patients with moderate to critical illness had significantly lower scores across all SF-36 subscales, particularly in the functionality, energy/fatigue, and general health subscales, than those with mild disease^19^. In the present study, analysis of the patients’ SF-36 scale scores at one month post-infection showed that patients in the ICU group had significantly lower scores in the following subscales than those in the outpatient and ward groups: physical function (p <0.001), physical role difficulty (p=0.001), energy vitality (p=0.028), social functionality (p=0.001), and general health perception (p <0.001). This disparity persisted at the 12-month follow-up, with the patients in the ICU group exhibiting lower scores in the physical function (p =0.001), physical role difficulty (p =0.025), and social functionality (p =0.031) subscales than the other groups. These findings underscore the sustained effects of severe COVID-19 on multiple aspects of health-related quality of life. Notably, the SF-36 quality of life subscales scores obtained at the first and third month follow-up timepoints in the present study are lower than the normal values for the general Turkish population ^20^. This observation is consistent with the findings of the study by Stefan et al., who reported that outpatients with symptoms that lasted longer than four weeks had had lower physical and mental health-related quality of life scores than the normal pre-pandemic population values^21^. Similarly, another observational cohort study of 101 patients indicated that at six weeks post-discharge, the participants exhibited significantly lower SF-36 scores in all subscales (except pain) than the general healthy population^22^. 

The PCFS scale is a quick and easy tool for identifying individuals who have not fully recovered from COVID-19. The scale focuses on limitations in daily life due to persistent symptoms of COVID-19. In a French observational, single-center, prospective study, 121 patients hospitalized for severe COVID-19 pneumonia were assessed using the PCFS scale over a median period of 125 days. The results indicated that only 44 patients (36.4%) recovered to their pre-COVID-19 functional status. Overall, 106 patients (88%) had PCFS scale scores of 0, 1, or 2, indicating minimal to mild functional limitations^23^. In another study, the PCFS scale was used to evaluate 239 patients at the 3-month and 6-month follow-up timepoints. The results demonstrated a significant improvement in functional status over time, with patients exhibiting better PCFS scores at 6 months than at 3 months^24^. In the present study, assessment of the patients during the pre-COVID period showed that 71.8% had a PCFS scale score of 0 or 1. However, this percentage decreased to 44.2% at the 6-month follow-up and increased slightly to 50% at 12 months. Although our findings align with previous reports that showed an improvement in PCFS scores over time, they also highlight that a substantial proportion of patients do not fully regain their pre-COVID functional status. Furthermore, our results revealed that the number of patients with a PCFS score of 0 at diagnosis and at the 12-month follow-up was significantly higher in the outpatient group than in the ICU and ward groups. Additionally, the frequency of PCFS grade 4 in the first month post-diagnosis was significantly lower in the outpatient group (p <0.001) than in other groups. In contrast, the frequency of grades 3 and 4 in the third month was lower the outpatient group than in other groups (p =0.002). These findings align with the results of a prospective cohort study conducted by Taboada et al., in which the PFCS scale was used to evaluate the functional status of 183 patients at 6 months post-discharge. The results of the study demonstrated a higher incidence of limitations in daily life (PCFS grades 2-4) in the ICU group patients (56.4%) than in the ward group (17.9%) (p <0.001), highlighting the greater functional impairment experienced by those who required intensive care during the acute COVID-19 phase^25^. An Egyptian study of 444 patients with COVID-19 who were treated in outpatient, ward, and ICU settings and evaluated in the post-COVID-19 period using the PCFS scale indicated that factors such as female sex, older age, presence of comorbid conditions, need for ICU admission, need for oxygen therapy, no history of receiving a seasonal flu vaccine, and smoking status are associated with higher PCFS scores. Notably, only 3% of the patients treated in the ICU reported no functional limitations^26^. Our findings are consistent with these observations, indicating higher PCFS scores and a greater prevalence of functional limitations in the ICU group. However, it is important to note that persistent functional and quality-of-life impairments observed in patients treated in the ICU may not be solely attributable to long COVID. Many of these symptoms, including fatigue, dyspnea, and cognitive dysfunction, are core features of Post-Intensive Care Syndrome (PICS), which can affect survivors of any critical illness^27^. 

Given the lack of a control group of patients without COVID-19 who are treated in the ICU, distinguishing between post-acute sequelae of COVID-19 (PASC) and PICS in the present study was challenging. Therefore, some of the observed long-term impairments may have multifactorial origins. Nevertheless, a notable strength of this study is the inclusion of outpatients, who are often underrepresented in literature on PASC. Most previous studies were primarily focused on hospitalized patients or patients treated in the ICU, leaving a knowledge gap in understanding the long-term outcomes of COVID-19 in patients with mild-to-moderate disease^14^ ^,^ ^16^ ^,^ ^28^. Results of the assessment of functional status and quality of life in outpatients in the present study provided valuable insights into this underrepresented population and highlighted the importance of long-term monitoring regardless of initial disease severity.

### Limitations

This study has several limitations. First, the sample sizes of this patient groups, particularly the ICU group, were relatively small. This may have reduced the statistical power of the subgroup analyses and limited the generalizability of the results to broader populations, particularly severely ill patients. Second, we did not include a control group of critically ill patients without COVID-19. This restricted our ability to differentiate between the long-term effects of COVID-19 and those associated with general critical illness or PICS, which is known to cause lasting functional and psychological impairments. Third, the symptoms were self-reported by patients during follow-up visits or telephone interviews. Therefore, the possibility of recall bias, particularly for symptoms assessed at the 6- and 12-month timepoints, cannot be completely ruled out. Moreover, participation in the follow-up assessments was voluntary. Therefore, it is possible that the patients who experienced persistent symptoms were more motivated to remain engaged in the study, which may have introduced a degree of selection bias. Fourth, although several important variables, such as age, sex, and comorbid conditions, were recorded and compared, the lack of multivariate analyses limited our ability to adjust for confounding factors that may have influenced the associations observed between disease severity and long-term outcomes. Finally, we lacked data on SARS-CoV-2 variants and vaccination status, both of which are being increasingly recognized as factors that affect the development and persistence of long-term COVID symptoms. These limitations should be considered when interpreting the conclusions drawn from our findings.

## CONCLUSIONS

ICU admission for COVID-19 often involves invasive interventions, such as mechanical ventilation, which may lead to persistent respiratory and functional impairments. In this study, assessment of functional impairment using the PCFS scale indicated that functional limitations caused by COVID-19 are most pronounced in the patients treated in the ICU. In addition, the analyses demonstrated that although these symptoms tend to improve over time, many persist even at the 12-month follow-up. In this study, quality of life, assessed using the SF-36, was significantly lower in the ICU patient group, especially in the physical functioning, role limitations, social functioning, and general health perception subscales. Although the patients treated as outpatients or in general wards demonstrated substantial recovery between the first and twelfth months, improvements in patients treated in the ICU were more limited. These findings suggest that long-term outcomes of COVID-19 may vary according to the initial care settings. In addition, the results of this study highlight the need for prolonged follow-up and targeted rehabilitation programs for patients diagnosed with COVID-19, particularly those who require intensive care. However, these conclusions should be interpreted with caution in light of the limitations of this study, including its sample size constraints, lack of multivariate analysis, and the absence of data on vaccination status and virus variants.

## References

[1] Han Q, Zheng B, Daines L, Sheikh A (2022). Long-Term Sequelae of COVID-19: A Systematic Review and Meta-Analysis of One-Year Follow-Up Studies on Post-COVID Symptoms. Pathogens.

[2] Carfì A, Bernabei R, Landi F, Gemelli Against COVID-19 Post-Acute Care Study Group (2020). Persistent Symptoms in Patients After Acute COVID-19. JAMA.

[3] Ahmed H, Patel K, Greenwood DC, Halpin S, Lewthwaite P, Salawu A (2020). Long-term clinical outcomes in survivors of severe acute respiratory syndrome (SARS) and Middle East respiratory syndrome coronavirus (MERS) outbreaks after hospitalisation or ICU admission: A systematic review and meta-analysis. J Rehabil Med.

[4] Soriano JB, Murthy S, Marshall JC, Relan P, Diaz JV, WHO Clinical Case Definition Working Group on Post-COVID-19 Condition (2022). A clinical case definition of post-COVID-19 condition by a Delphi consensus. Lancet Infect Dis.

[5] Nalbandian A, Desai AD, Wan EY (2023). Post-COVID-19 Condition. Annu Rev Med.

[6] Akbarialiabad H, Taghrir MH, Abdollahi A, Ghahramani N, Kumar M, Paydar S (2021). Long COVID, a comprehensive systematic scoping review. Infection.

[7] World Health Organization (2025). WHO coronavirus (COVID-19) dashboard.

[8] Sayan M, Arikan A, Isbilen M (2022). Circulating Dynamics of SARS-CoV-2 Variants between April 2021 and February 2022 in Turkey. Can J Infect Dis Med Microbiol.

[9] Akçeşme FB, Köprülü TK, Erkal B, İş Ş, Keskin BC, Akçeşme B (2022). Tracking the circulating SARS-CoV-2 variants in Turkey: complete genome sequencing and molecular characterization of 1000 SARS-CoV-2 samples. BioRxiv.

[10] (2020). COVID-19 rapid guideline: managing the long-term effects of COVID-19.

[11] Klok FA, Boon GJAM, Barco S, Endres M, Geelhoed JJM, Knauss S, Rezek SA (2020). The Post-COVID-19 Functional Status scale: a tool to measure functional status over time after COVID-19. Eur Respir J.

[12] Ware JE, Sherbourne CD (1992). The MOS 36-item short-form health survey (SF-36). I. Conceptual framework and item selection. Med Care.

[13] Lopez-Leon S, Wegman-Ostrosky T, Perelman C, Sepulveda R, Rebolledo PA, Cuapio A (2021). More than 50 Long-term effects of COVID- 19: a systematic review and meta-analysis. Sci Rep.

[14] Huang C, Huang L, Wang Y, Li X, Ren L, Gu X (2023). 6-month consequences of COVID-19 in patients discharged from hospital: a cohort study. The Lancet.

[15] Peghin M, Palese A, Venturini M, De Martino M, Gerussi V, Graziano E (2021). Post-COVID-19 symptoms 6 months after acute infection among hospitalized and non-hospitalized patients. Clin Microbiol Infect.

[16] Dryden M, Mudara C, Vika C, Blumberg L, Mayet N, Cohen C (2022). Post-COVID-19 condition 3 months after hospitalisation with SARS-CoV-2 in South Africa: a prospective cohort study. Lancet Glob Health.

[17] Khoddami SM, Aghadoost S, Aghajanzadeh M, Molazeinal Y (2023). The Health-related Quality of Life and Voice Handicap Index in Recovered COVID-19 Patients in Comparison to Healthy Subjects. J Voice.

[18] McFann K, Baxter BA, LaVergne SM, Stromberg S, Berry K, Tipton M (2021). Quality of Life (QoL) Is Reduced in Those with Severe COVID-19 Disease, Post-Acute Sequelae of COVID-19, and Hospitalization in United States Adults from Northern Colorado. Int J Environ Res Public Health.

[19] van den Borst B, Peters JB, Brink M, Schoon Y, Bleeker-Rovers CP, Schers H (2021). Comprehensive Health Assessment 3 Months After Recovery From Acute Coronavirus Disease 2019 (COVID-19). Clin Infect Dis.

[20] Demiral Y, Ergor G, Unal B, Semin S, Akvardar Y, Kivircik B (2006). Normative data and discriminative properties of short form 36 (SF-36) in Turkish urban population. BMC Public Health.

[21] Malesevic S, Sievi NA, Baumgartner P, Roser K, Sommer G, Schmidt D (2023). Impaired health-related quality of life in long-COVID syndrome after mild to moderate COVID-19. Sci Rep.

[22] van der Sar-van der Brugge S, Talman S, Boonman-de Winter L, de Mol M, Hoefman E, van Etten RW (2021). Pulmonary function and health-related quality of life after COVID-19 pneumonia. Respir Med.

[23] Benkalfate N, Eschapasse E, Georges T, Leblanc C, Dirou S, Melscoet L (2022). Evaluation of the Post-COVID-19 Functional Status (PCFS) Scale in a cohort of patients recovering from hypoxemic SARS-CoV-2 pneumonia. BMJ Open Respir Res.

[24] Vaes AW, Goërtz YMJ, Van Herck M, Machado FVC, Meys R, Delbressine JM (2021). Recovery from COVID-19: a sprint or marathon? 6-month follow-up data from online long COVID-19 support group members. ERJ Open Res.

[25] Taboada M, Cariñena A, Moreno E, Rodríguez N, Domínguez MJ, Casal A (2021). Post-COVID-19 functional status six-months after hospitalization. J Infect.

[26] Mohamed Hussein AA, Saad M, Zayan HE, Abdelsayed M, Moustafa M, Ezzat AR (2021). Post-COVID-19 functional status: Relation to age, smoking, hospitalization, and previous comorbidities. Ann Thorac Med.

[27] Fleischmann-Struzek C, Joost FEA, Pletz MW, Weiß B, Paul N, Ely EW (2024). How are Long-Covid, Post-Sepsis-Syndrome and Post-Intensive-Care-Syndrome related? A conceptional approach based on the current research literature. Crit Care.

[28] Mandal S, Barnett J, Brill SE, Brown JS, Denneny EK, Hare SS (2021). Long-COVID': a cross-sectional study of persisting symptoms, biomarker and imaging abnormalities following hospitalisation for COVID-19. Thorax.

