# Supplementary material for: Follow-up evaluation of long COVID syndrome in patients with SARS-CoV-2 infection
Source: Rev Soc Bras Med Trop. 2025 Aug 8;58:e0046-2025. doi: 10.1590/0037-8682-0046-2025 (PMC12333617; doi:10.1590/0037-8682-0046-2025)
Supplement: Supplementary file 1 [file 1678-9849-rsbmt-58-e0046-2025-supp1.pdf]

SUPLEMANTARY TABLE 1: Comparison of symptoms at the time of diagnosis at the first, third, sixth, and twelfth months post-diagnosis.

Diagnostic findings	At diagnosis	1st month	3rd month	6th month	12th month
	n (%)	n (%)	n (%)	n (%)	n (%)
**Fever**					
Outpatient (n=67)	30 (44.8)	1 (1.5)	-	-	-
General ward (n=47)	32 (68.1)	3 (6.4)	-	-	-
Intensive care unit (n=17)	12 (70.6)	1 (5.9)	-	-	-
p-value	0.021*	0.363			
Weakness and fatigue					
Outpatient (n=67)	54 (80.6)	31 (46.3)	23 (34.3)	16(23.9)	9 (13.4)
General ward (n=47)	45 (95.7)	29 (61.7)	18 (38.3)	10(21.3)	6 (12.8)
Intensive care unit (n=17)	14 (82.4)	14 (82.4)	11 (64.7)	8 (47.1)	6 (35.3)
p-value	0.061	0.018*	0.071	0.099	0.067
Easy fatigue during exertion					
Outpatient (n=67)	41 (61.2)	45 (67.2)	30 (44.8)	15 (22.4)	13 (19.4)
General ward (n=47)	37 (78.7)	38 (80.9)	22 (46.8)	22 (46.8)	21 (44.7)
Intensive care unit (n=17)	14 (82.4)	15 (88.2)	14 (82.4)	13 (76.5)	14 (82.4)
p-value	0.066	0.100	0.018*	<0.001**	<0.001**
Cough					
Outpatient (n=67)	41 (61.2)	15 (22.4)	4 (6.0)	2 (3.0)	1 (1.5)
General ward (n=47)	38 (80.9)	11 (23.4)	3 (6.4)	3 (6.4)	1 (2.1)
Intensive care unit (n=17)	12 (70.6)	8 (47.1)	4 (23.5)	1 (5.9)	1 (5.9)
p-value	0.080	0.103	0.054	0.668	0.555
Dyspnea					
Outpatient (n=67)	25 (37.3)	12 (17.9)	3 (4.5)	1 (1.5)	-
General ward (n=47)	27 (57.4)	12 (25.5)	5 (10.6)	4 (8.5)	-
Intensive care unit (n=17)	14 (82.4)	11 (64.7)	6 (35.3)	3 (17.6)	2 (11.8)
p-value	0.002**	<0.001**	0.001**	0.032*	0.001**
Myalgia					
Outpatient (n=67)	49 (73.1)	21 (31.3)	14 (20.9)	5 (7.5)	4 (6.0)
General ward (n=47)	33 (70.2)	18 (38.3)	7 (14.9)	6 (12.8)	3 (6.4)
Intensive care unit (n=17)	11 (64.7)	6 (35.3)	3 (17.6)	3 (17.6)	2 (11.8)
p-value	0.783	0.741	0.715	0.405	0.691
Arthralgia					
Outpatient (n=67)	41 (61.2)	15 (22.4)	8 (11.9)	2 (3.0)	4 (6.0)
General ward (n=47)	27 (57.4)	16 (34.0)	6 (12.9)	4 (8.5)	5 (10.6)
Intensive care unit (n=17)	8 (47.1)	5 (29.4)	3 (17.6)	3 (17.6)	-
p-value	0.571	0.383	0.821	0.088	0.304

Continue...

**Palpitation**					
Outpatient (n=67)	23 (34.3)	14 (20.9)	7 (10.4)	2 (3.0)	3 (4.5)
General ward (n=47)	13 (27.7)	7 (14.9)	4 (8.5)	6 (12.8)	4 (8.5)
Intensive care unit (n=17)	6 (35.3)	4 (23.5)	3 (17.6)	2 (11.8)	5 (29.4)
p-value	0.720	0.639	0.577	0.121	0.006**
**Headache**					
Outpatient (n=67)	41 (61.2)	16 (23.9)	9 (13.4)	2 (3.0)	1 (1.5)
General ward (n=47)	24 (51.1)	7 (14.9)	6 (12.8)	4 (8.5)	1 (2.1)
Intensive care unit (n=17)	7 (41.2)	2 (11.8)	2 (11.8)	1 (5.9)	1 (5.9)
p-value	0.266	0.346	0.982	0.432	0.555
**Sleep disturbances**					
Outpatient (n=67)	31 (46.3)	13 (19.4)	8 (11.9)	3 (4.5)	-
General ward (n=47)	23 (48.9)	8 (17.0)	5 (10.6)	5 (10.6)	2 (4.3)
Intensive care unit (n=17)	10 (58.8)	10 (58.8)	4 (23.5)	7 (41.2)	1 (5.9)
p-value	0.652	0.001**	0.374	<0.001**	0.186
**Forgetfulness**					
Outpatient (n=67)	24 (35.8)	15 (22.4)	13 (19.4)	9 (13.4)	2 (3.0)
General ward (n=47)	16 (34.0)	9 (19.1)	10 (21.3)	3 (6.4)	3 (6.4)
Intensive care unit (n=17)	8 (47.1)	8 (47.1)	6 (35.3)	6 (35.3)	1 (5.9)
p-value	0.622	0.062	0.365	0.012*	0.668
**Difficulty concentrating**					
Outpatient (n=67)	24 (35.8)	13 (19.4)	6 (9.0)	6 (9.0)	2 (3.0)
General ward (n=47)	10 (21.3)	7 (14.9)	2 (4.3)	2 (4.3)	2 (4.3)
Intensive care unit (n=17)	5 (29.4)	6 (35.3)	3 (17.6)	2 (11.8)	-
p-value	0.247	0.194	0.227	0.512	0.682
**Loss of appetite**					
Outpatient (n=67)	35 (52.2)	7 (10.4)	3 (4.5)	4 (6.0)	1 (1.5)
General ward (n=47)	31 (66.0)	8 (17.0)	2 (4.3)	-	2 (4.3)
Intensive care unit (n=17)	11 (64.7)	5 (29.4)	3 (17.6)	1 (5.9)	1 (5.9)
p-value	0.297	0.139	0.103	0.234	0.538
**Loss of smell**					
Outpatient (n=67)	51 (76.1)	22 (32.8)	16 (23.9)	10 (14.9)	3 (4.5)
General ward (n=47)	23 (48.9)	8 (17.0)	2 (4.3)	1 (2.1)	-
Intensive care unit (n=17)	5 (29.4)	2 (11.8)	1 (5.9)	1 (5.9)	-
p-value	<0.001**	0.066	0.008**	0.058	0.231
**Loss of taste**					
Outpatient (n=67)	46 (68.7)	15 (22.4)	9 (13.4)	7 (10.4)	2 (3.0)
General ward (n=47)	25 (53.2)	8 (17.0)	2 (4.3)	1 (2.1)	-
Intensive care unit (n=17)	5 (29.4)	1 (5.9)	1 (5.9)	1 (5.9)	-
p-value	0.010*	0.279	0.218	0.221	0.379

Continue...

**Diarrhea**					
Outpatient (n=67)	12 (17.9)	4 (6.0)	-	-	-
General ward (n=47)	13 (27.7)	-	1 (2.1)	-	-
Intensive care unit (n=17)	3 (17.6)	-	-	-	-
p-value	0.422	0.139	0.406		
**Skin-related symptoms**					
Outpatient (n=67)	6 (9.0)	-	-	-	-
General ward (n=47)	-	-	-	-	-
Intensive care unit (n=17)	-	-	-	-	-
p-value	0.050				
**Vision problems**					
Outpatient (n=67)	3 (4.5)	1 (1.5)	1 (1.5)	-	1 (1.5)
General ward (n=47)	-	-	-	-	-
Intensive care unit (n=17)	-	-	-	-	-
p-value	0.231	0.618	0.618		0.618

* p-value <0.05, **p-value <0.001, chi-square and Fisher's exact test.
