# Supplementary material for: Follow-up evaluation of long COVID syndrome in patients with SARS-CoV-2 infection
Source: Rev Soc Bras Med Trop. 2025 Aug 8;58:e0046-2025. doi: 10.1590/0037-8682-0046-2025 (PMC12333617; doi:10.1590/0037-8682-0046-2025)
Supplement: Supplementary file 2 [file 1678-9849-rsbmt-58-e0046-2025-supp2.pdf]

SUPLEMANTARY TABLE 2: Summary of the PCFS scale scores of the patient groups.

	Total	Outpatient (n=67)	General ward (n=47)	Intensive care unit (n=17)	p value[a]
	n(%)	n(%)	n(%)	n(%)	
**Pre-COVID-19 PCFS scale score**					
0	94 (71.8)	59 (88.1)	30 (63.8)	5 (29.4)	
1	-	-	-	-	
2	34 (26)	8 (11.9)	15 (31.9)	11 (64.7)	**<0.001****
3	3 (2.2)	-	2(4.3)	1(5.9)	
4		-	-	-	
**PCFS scale score at the time of diagnosis**					
0	2 (1.5)	2 (3.0)	-	-	
1	10 (7.6)	8 (11.9)	2 (4.3)	-	
2	11 (8.4)	10 (14.9)	1 (2.1)	-	**<0.001****
3	88 (67.2)	42 (62.7)	37 (78.7)	9 (52.9)	
4	20 (15.3)	5 (7.5)	7 (14.9)	8 (47.1)	
**PCFS scale score at 1 month post-diagnosis**					
0	15 (11.5)	7 (10.4)	7 (14.9)	1 (5.9)	
1	4 (3.1)	4 (6.0)	-	-	
2	17 (13)	11 (16.4)	6 (12.8)	-	**<0.001****
3	85 (64.9)	44 (65.7)	32 (68.1)	9 (52.9)	
4	10 (7.6)	1 (1.5)	2 (4.3)	7 (41.2)	
**PCFS scale score at 3 months post-diagnosis**					
0	32 (24.6)	17 (25.8)	14 (29.8)	1 (5.9)	
1	10 (7.7)	9 (13.6)	1 (2.1)	-	
2	30 (23.1)	17 (25.8)	9 (19.1)	4 (23.5)	**0.003****
3	55 (42.3)	23 (34.8)	22 (46.8)	10 (58.8)	
4	2 (1.5)	-	-	2 (11.8)	
D	1 (0.8)	-	1(2.1)	-	
**PCFS scale score at 6 months post-diagnosis**					
0	45 (34.9)	26 (40.0)	17 (36.2)	2 (11.8)	
1	12 (9.3)	9 (13.8)	2 (4.3)	1 (5.9)	
2	37 (28.7)	17 (26.2)	15 (31.9)	5 (29.4)	0.121
3	32 (24.8)	13 (20.0)	11 (23.4)	8 (47.1)	
4	2 (1.6)	-	1 (2.2)	1 (5.9)	
D	1 (0.8)	-	1(2.1)	-	
**PCFS scale score at 12 months post-diagnosis**					
0	54 (42.2)	36 (55.4)	16 (34.8)	2 (11.8)	
1	10 (7.8)	7 (10.8)	2 (4.3)	1 (5.9)	
2	42 (32.8)	16 (24.6)	18 (39.1)	8 (47.1)	**0.003****
3	19 (14.8)	6(9.2)	7(15.2)	6(35.3)	
4	-	-	-	-	
D	3 (2.3)	-	3(6.5)	-	

* p-value<0.05, **p-value<0.001. **a:** chi-square and Fisher's exact test; **PCFS:** Post-COVID-19 Functional Status; **D:** PCFS Grade 5 (death). Included in the analysis.
